# Supplementary material for: Robust single nucleus RNA sequencing reveals depot-specific cell population dynamics in adipose tissue remodeling during obesity
Source: bioRxiv. 2024 Aug 12:2024.04.08.588525. Originally published 2024 Apr 8. Preprint. [Version 2] doi: 10.1101/2024.04.08.588525 (PMC11030456; doi:10.1101/2024.04.08.588525)
Supplement: Supplement 1 [file NIHPP2024.04.08.588525v2-supplement-1.pdf]

bioRxiv preprint doi: https://doi.org/10.1101/2024.04.08.588525; this version posted August 12, 2024. The copyright holder for this preprint (which was not certified by peer review) is the author/funder, who has granted bioRxiv a license to display the preprint in perpetuity. It is made available under aCC-BY 4.0 International license.

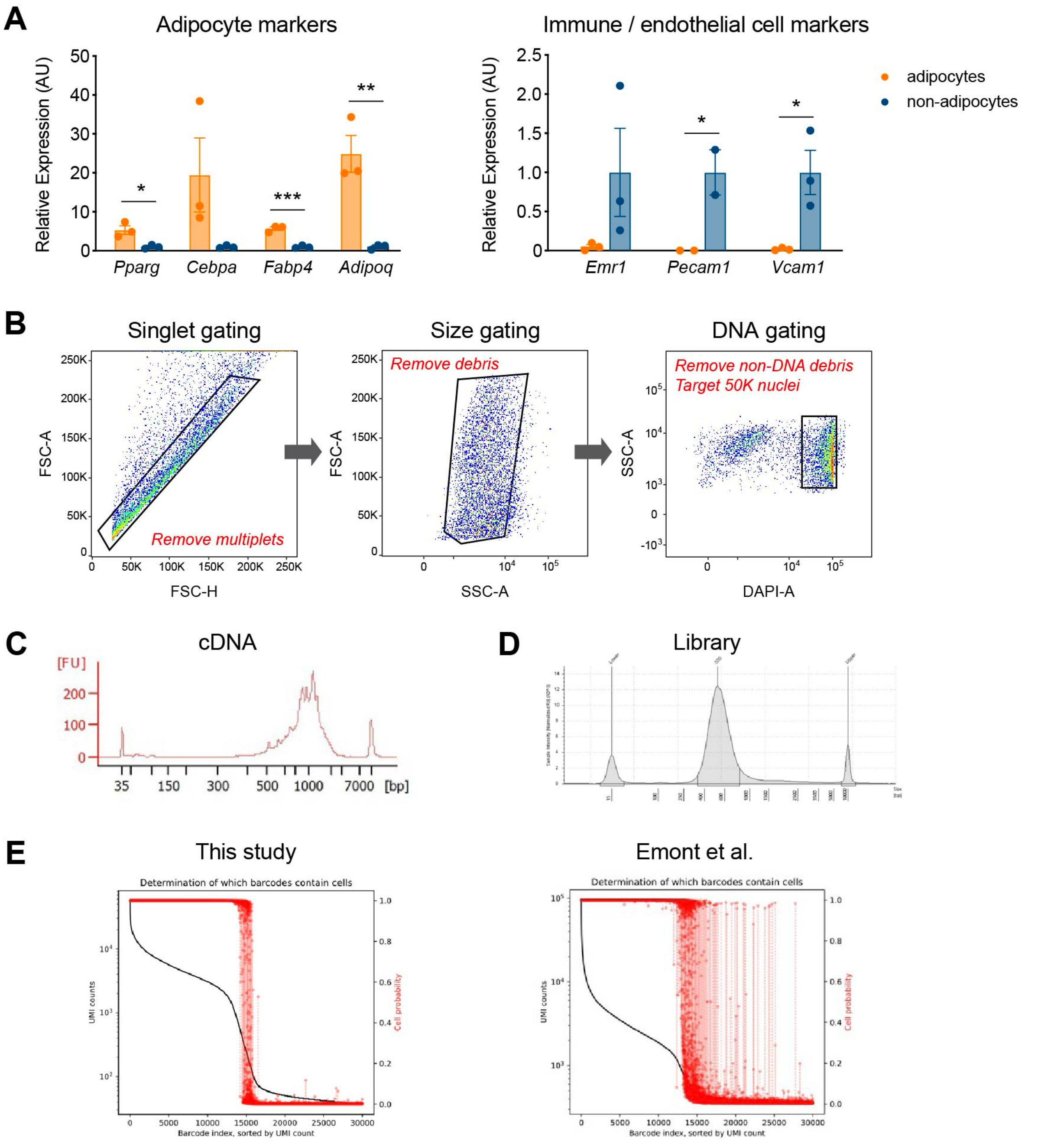


**Figure S1. Precise and robust gene expression profiling by our optimized snRNA-seq protocol. (A)** Gene expression analysis of adipocyte and immune/endothelial cell markers in adipocyte and non-adipocyte nuclei ($n$=3, each) from eWAT of NuTRAP mice, utilizing the optimized protocol with VRC. Data are presented as mean ± SEM, and statistical analysis was performed using Student's $t$ test. * $p$ <0.05, ** $p$ <0.01, *** $p$ <0.001. **(B)** Sequential gating strategies for fluorescence-activated nucleus sorting (FANS) used to sort all Hoechst-positive

bioRxiv preprint doi: https://doi.org/10.1101/2024.04.08.588525; this version posted August 12, 2024. The copyright holder for this preprint (which was not certified by peer review) is the author/funder, who has granted bioRxiv a license to display the preprint in perpetuity. It is made available under aCC-BY 4.0 International license.

nuclei from adipose tissue for snRNA-seq. **(C-D)** Exemplary profiles of **(C)** cDNA and **(D)** sequencing library fragment size distribution, assessed by Agilent Bioanalyzer and TapeStation, respectively. **(E)** Levels of ambient RNA in the samples of this study (left) and Emont et al. (GSE176171, right), examined using CellBender. The plots describe both the rank-ordered UMI counts per droplet (represented by a black line) and the probabilities of each droplet containing a cell (indicated by red dots with dashed lines).

bioRxiv preprint doi: https://doi.org/10.1101/2024.04.08.588525; this version posted August 12, 2024. The copyright holder for this preprint (which was not certified by peer review) is the author/funder, who has granted bioRxiv a license to display the preprint in perpetuity. It is made available under aCC-BY 4.0 International license.

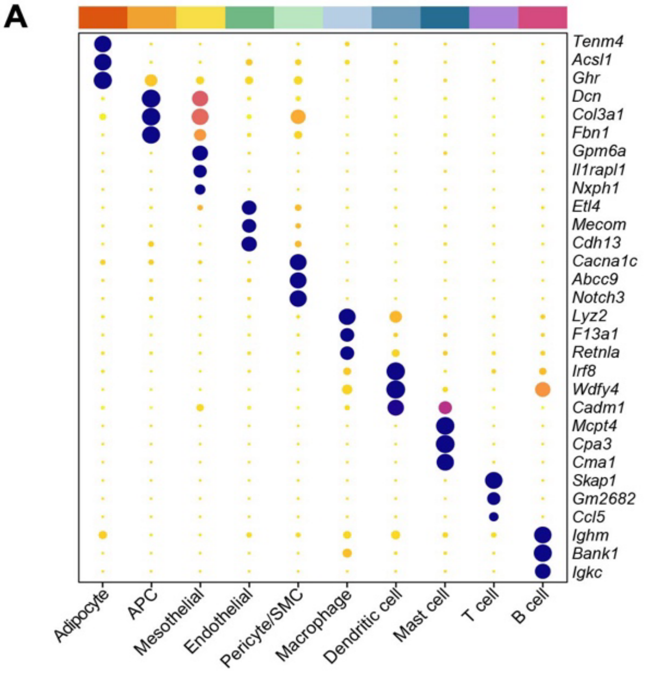


B

| | This study | Emont et al. | Sávári et al. |
|---|---|---|---|
| | nuclei no. (%) | nuclei no. (%) | nuclei no. (%) |
| Adipocyte | 10760 (20.2) | 39934 (21.7) | 4604 (24.3) |
| APC | 14061 (26.3) | 51227 (27.8) | 5172 (27.3) |
| Mesothelial | 1982 (3.7) | 14947 (8.1) | 790 (4.2) |
| Endothelial | 6747 (12.6) | 6772 (3.7) | NA |
| Pericyte/SMC | 1830 (3.4) | 860 (0.5) | NA |
| Macrophage | 15505 (29.0) | 65502 (35.5) | 7618 (40.3) |
| Dendritic cell | 359 (0.7) | 1572 (0.9) | 737 (3.9) |
| Mast cell | 181 (0.3) | 311 (0.2) | NA |
| T cell | 1361 (2.6) | 1960 (1.1) | NA |
| B cell | 609 (1.14) | 1202 (0.7) | NA |
| Total | 53395 (100) | 184287 (100) | 18921 (100) |

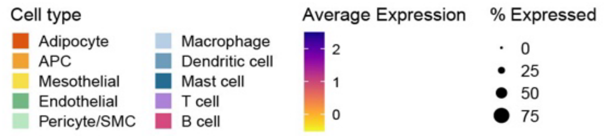


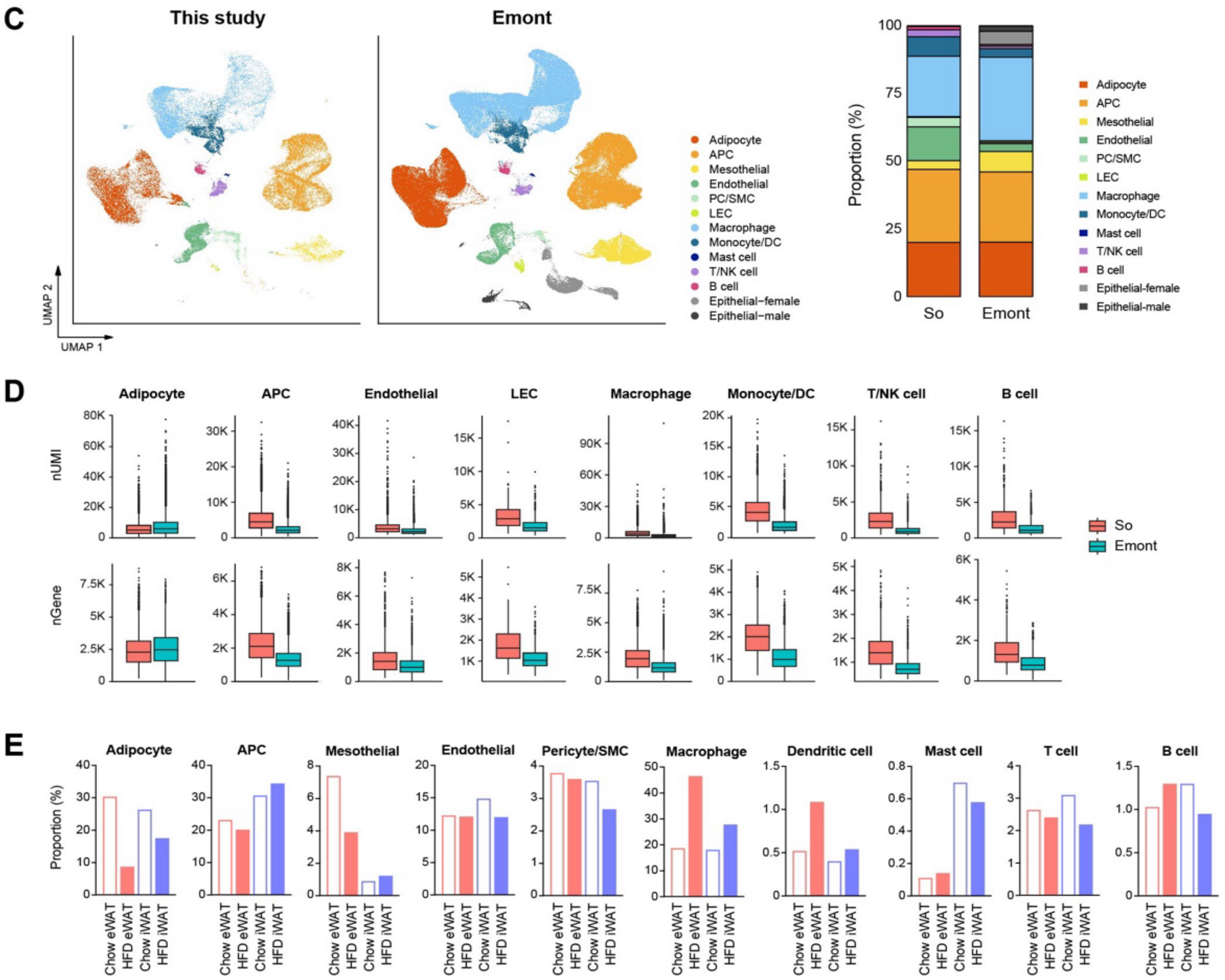

bioRxiv preprint doi: https://doi.org/10.1101/2024.04.08.588525; this version posted August 12, 2024. The copyright holder for this preprint (which was not certified by peer review) is the author/funder, who has granted bioRxiv a license to display the preprint in perpetuity. It is made available under aCC-BY 4.0 International license.

**Figure S2. Profile of our single nucleus atlas encompassing diverse cell populations. (A)** Dot plots showing average scaled expression of the top three cell type marker genes in each cell population in mouse adipose tissue. **(B-D)** Comparison of mouse adipose tissue snRNA-seq data from this study with data from previous snRNA-seq studies. **(B)** The absolute numbers and relative proportions of nuclei recovered for each cell type. **(C)** Integration of all cell types from this study with those from Emont *et al.* Bar graphs on the right side display the relative proportions of individual cell types in each dataset. **(D)** Comparison of UMI counts and genes detected per nucleus by cell type between this study and Emont *et al.* **(E)** Bar graphs showing the relative proportions of individual cell types by fat depot and diet in this study.

bioRxiv preprint doi: https://doi.org/10.1101/2024.04.08.588525; this version posted August 12, 2024. The copyright holder for this preprint (which was not certified by peer review) is the author/funder, who has granted bioRxiv a license to display the preprint in perpetuity. It is made available under aCC-BY 4.0 International license.

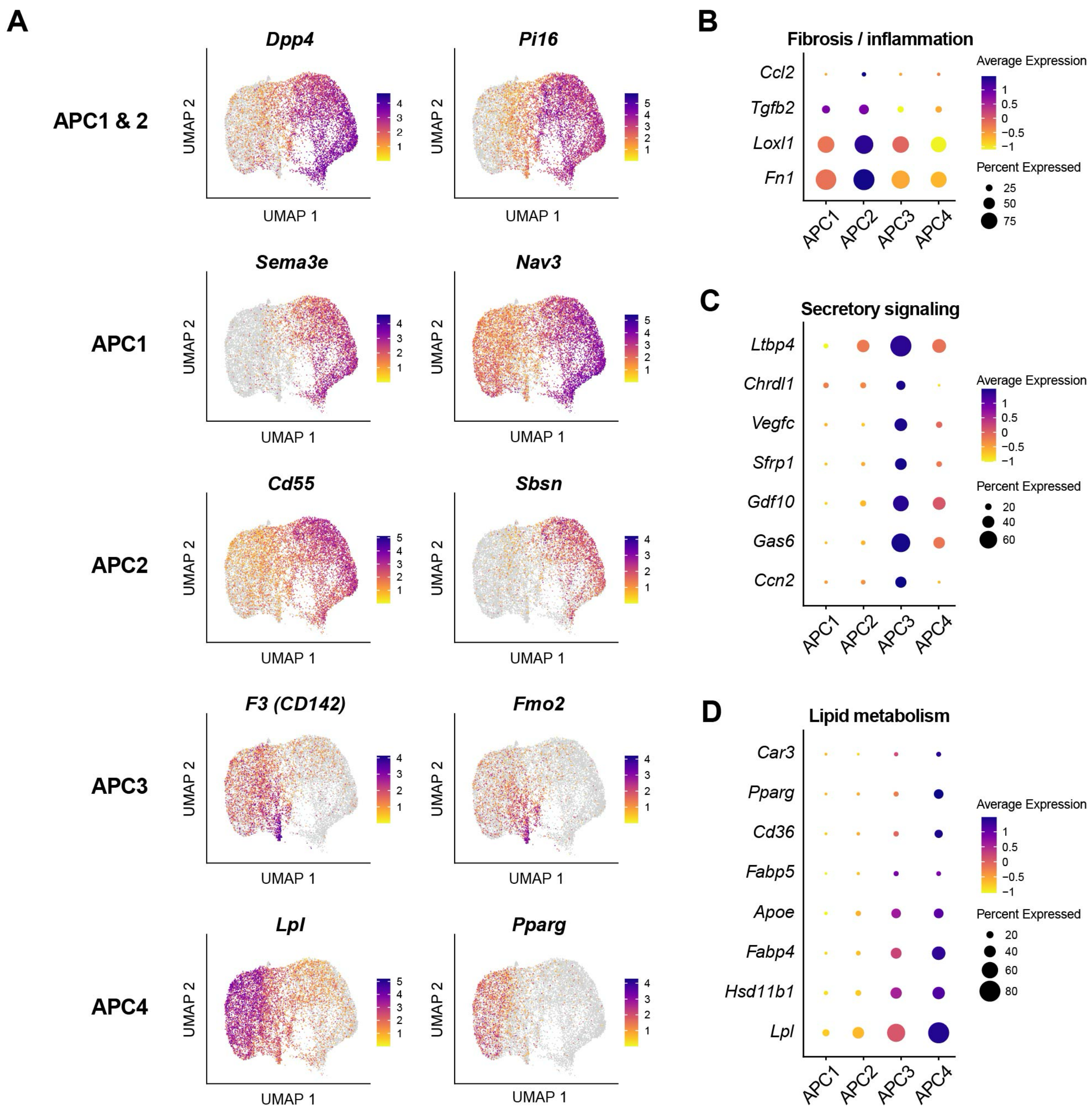


**Figure S3. Gene expression profiles of APC subpopulations indicating distinct functional states. (A)** UMAP visualization of marker genes for APC subpopulations. **(B-D)** Dot plots showing the expression of genes associated with fibrosis/inflammation **(B)**, secretory signaling **(C)**, and lipid metabolism **(D)** across APC subpopulations.

bioRxiv preprint doi: https://doi.org/10.1101/2024.04.08.588525; this version posted August 12, 2024. The copyright holder for this preprint (which was not certified by peer review) is the author/funder, who has granted bioRxiv a license to display the preprint in perpetuity. It is made available under aCC-BY 4.0 International license.

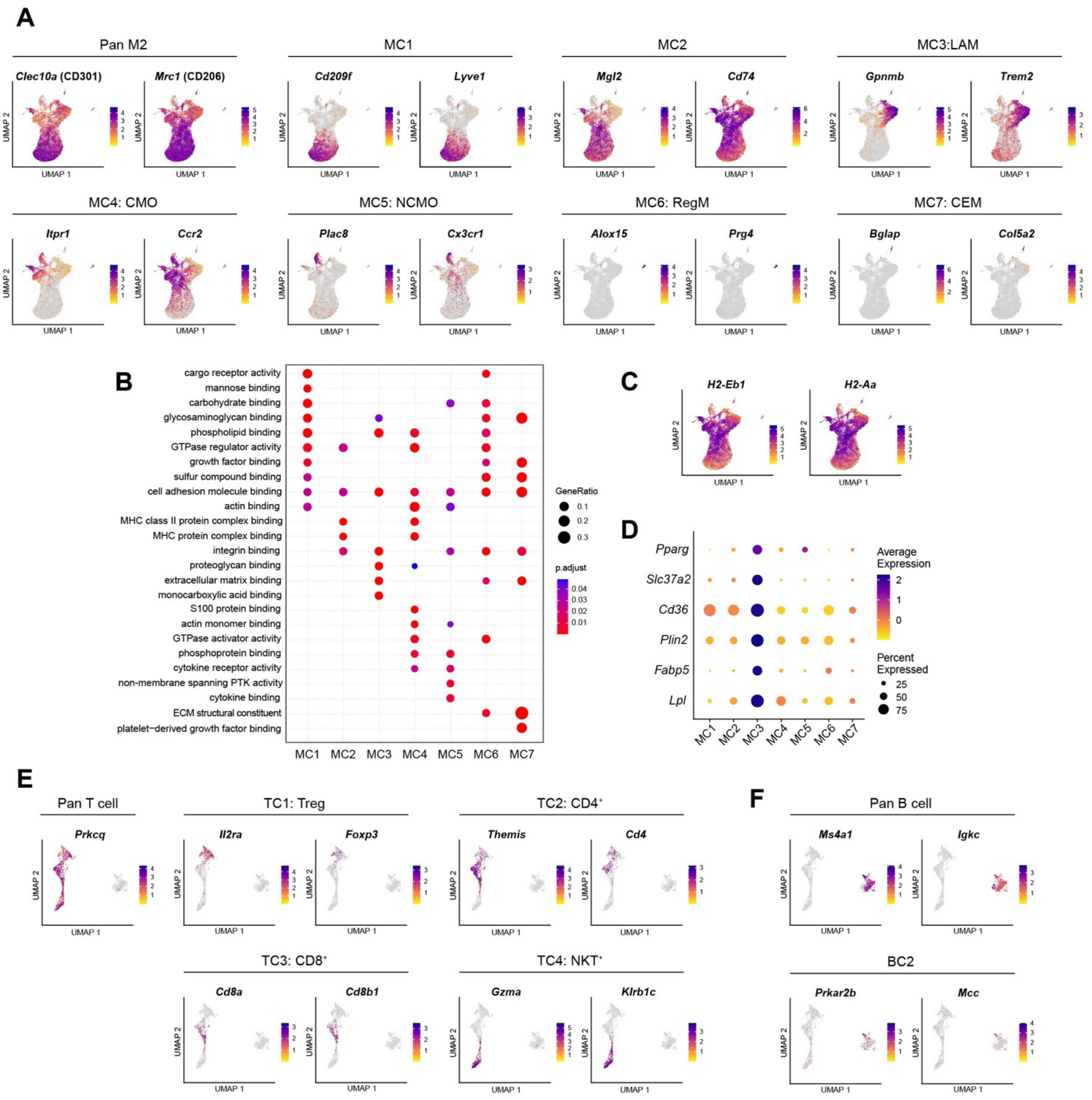


**Figure S4. Characterization of diverse immune cell subpopulations. (A)** UMAP visualization of marker genes for macrophage/monocyte subpopulations. **(B)** Dot plot illustrating pathways enriched within the top 100 marker genes of macrophage/monocyte subpopulations, as determined by clusterProfiler. **(C)** UMAP visualization of major histocompatibility complex (MHC) genes in macrophage/monocyte subpopulations. **(D)** Dot plot showing the expression of genes associated with lipid metabolism across macrophage/monocyte subpopulations. **(E)** UMAP visualization of marker genes for lymphocyte subpopulations.

bioRxiv preprint doi: https://doi.org/10.1101/2024.04.08.588525; this version posted August 12, 2024. The copyright holder for this preprint (which was not certified by peer review) is the author/funder, who has granted bioRxiv a license to display the preprint in perpetuity. It is made available under aCC-BY 4.0 International license.

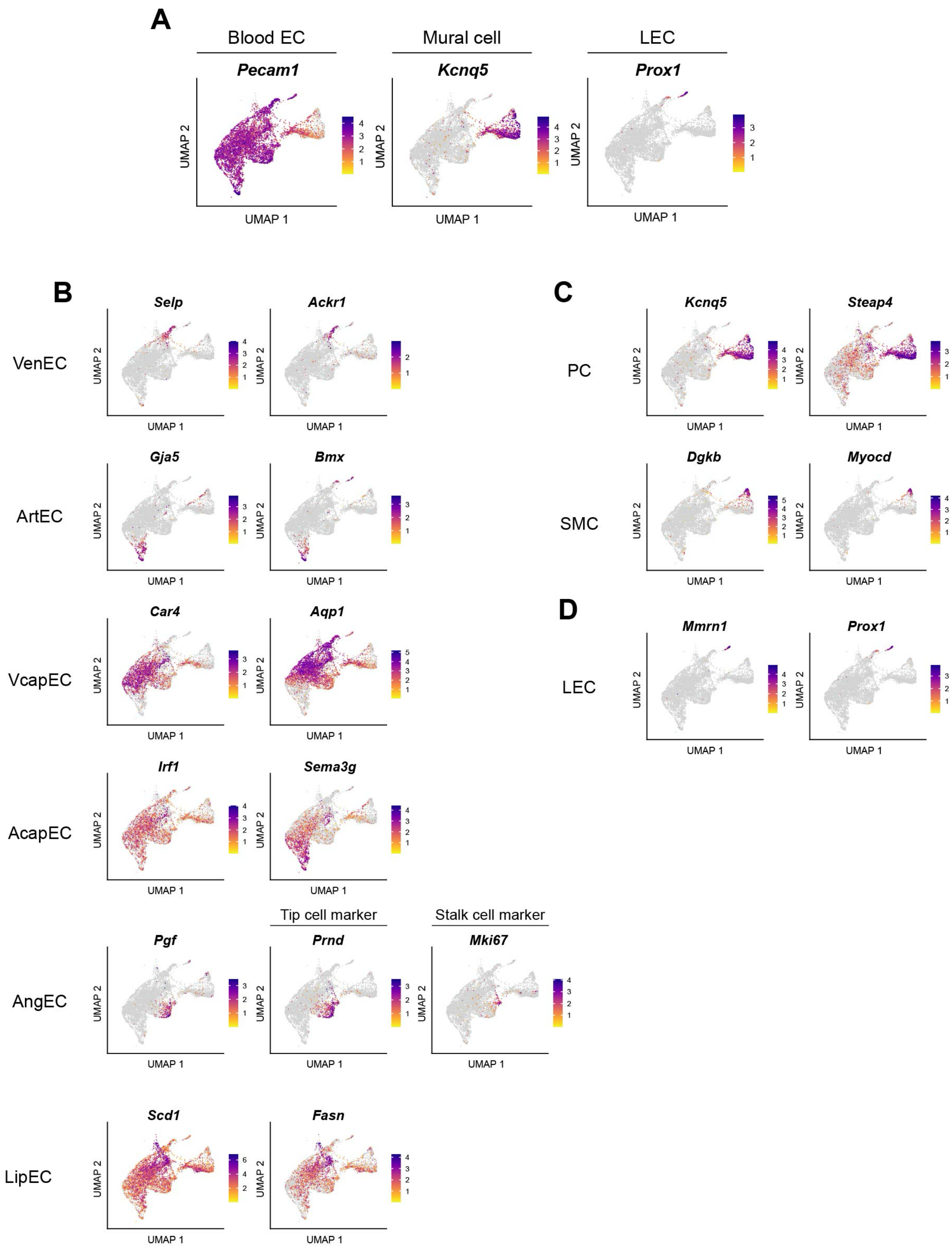

bioRxiv preprint doi: https://doi.org/10.1101/2024.04.08.588525; this version posted August 12, 2024. The copyright holder for this preprint (which was not certified by peer review) is the author/funder, who has granted bioRxiv a license to display the preprint in perpetuity. It is made available under aCC-BY 4.0 International license.

**Figure S5. Characterization of diverse vascular cell subpopulations. (A)** UMAP visualization using *Pecam1* for blood EC, *Kcnq5* for mural cells, and *Prox1* for LEC within vascular cells. **(B-D)** UMAP visualization of marker genes for **(B)** blood EC subpopulations. **(C)** mural cell subpopulations, and **(D)** LEC subpopulation.

bioRxiv preprint doi: https://doi.org/10.1101/2024.04.08.588525; this version posted August 12, 2024. The copyright holder for this preprint (which was not certified by peer review) is the author/funder, who has granted bioRxiv a license to display the preprint in perpetuity. It is made available under aCC-BY 4.0 International license.

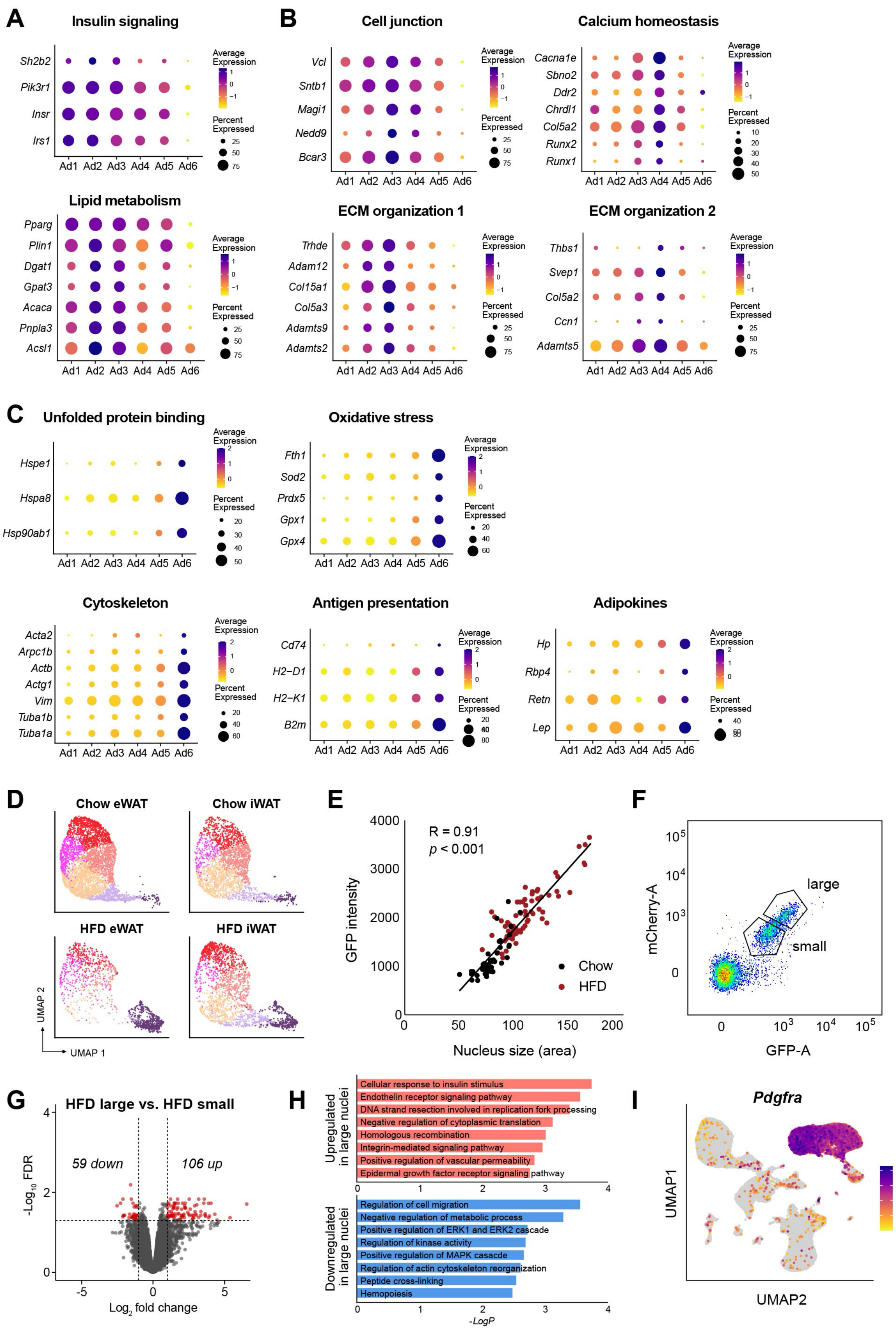

bioRxiv preprint doi: https://doi.org/10.1101/2024.04.08.588525; this version posted August 12, 2024. The copyright holder for this preprint (which was not certified by peer review) is the author/funder, who has granted bioRxiv a license to display the preprint in perpetuity. It is made available under aCC-BY 4.0 International license.

**Figure S6. Identification of functional attributes of adipocyte subpopulations. (A-C)** Dot plots representing the expression of genes linked to distinct biological processes enriched in different adipocyte subpopulations: **(A)** for Ad1-2, **(B)** for Ad3-4, and **(C)** for Ad6. **(D)** UMAP plots of adipocyte subpopulations by fat depot and diet. **(E)** Correlation between nucleus size and GFP intensity in adipocyte nuclei isolated from adipose tissues of chow-fed (black) or HFD-fed (red) Ad-NuTRAP mice. *P* value was calculated using an F-test with the null hypothesis that the slope is equal to 0. **(F)** Sorting criteria for gating adipocyte nuclei isolated from iWAT of HFD-fed NuTRAP mice into small and large nuclei. **(G)** Volcano plot showing statistical significance ($-\log_{10}$FDR values) versus fold change of gene expression between large and small adipocyte nuclei from iWAT of HFD-fed mice. Red dots represent genes that were differentially expressed between groups with an FDR < 0.05 and $|\log_2$ fold change$| > 1$. **(H)** Bar graphs illustrating the top GO terms (GO: BP) enriched in upregulated (top) and downregulated (bottom) genes in large versus small adipocyte nuclei. **(I)** UMAP visualization of an APC marker gene, *Pdgfra*.

bioRxiv preprint doi: https://doi.org/10.1101/2024.04.08.588525; this version posted August 12, 2024. The copyright holder for this preprint (which was not certified by peer review) is the author/funder, who has granted bioRxiv a license to display the preprint in perpetuity. It is made available under aCC-BY 4.0 International license.

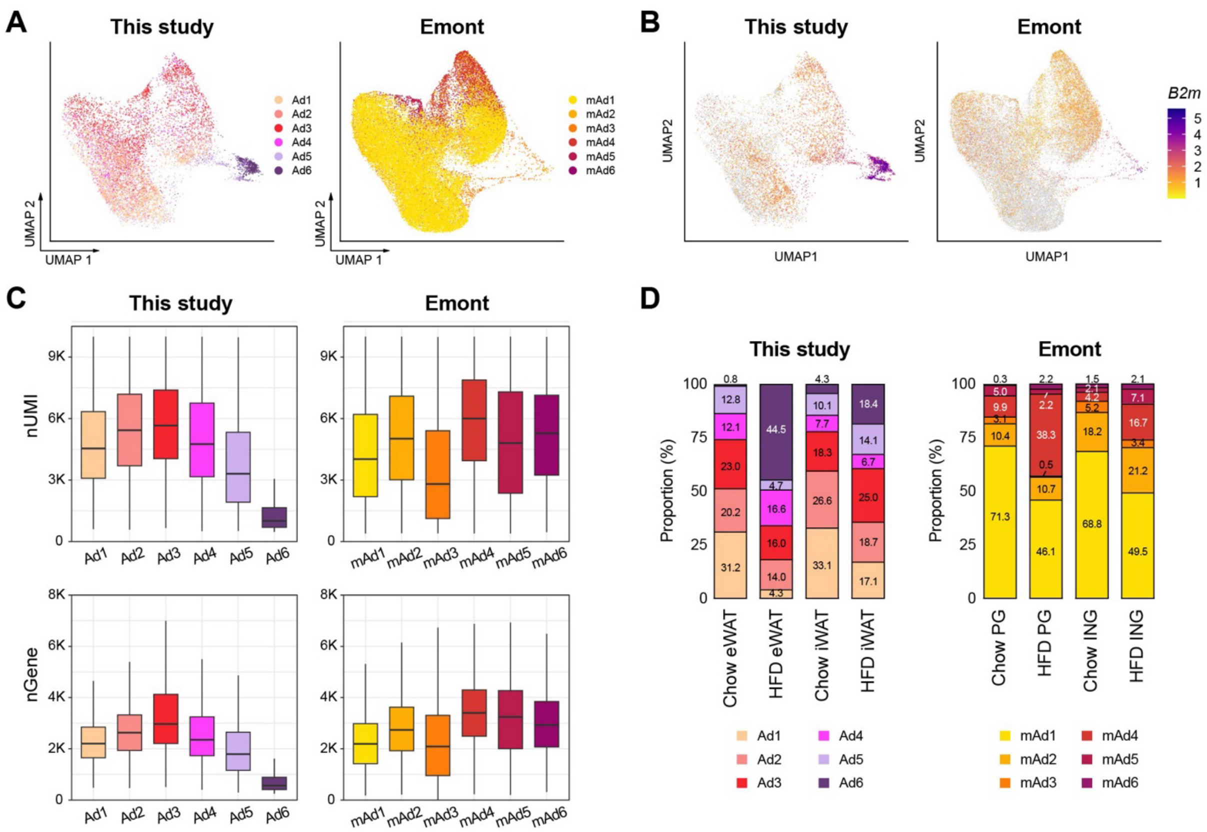


**Figure S7. Comparison of adipocyte subpopulations identified in this study versus Emont et al. (A)** UMAP visualization of integrated adipocyte nuclei from this study and Emont et al. **(B)** B2M gene expression in adipocyte nuclei from each dataset. **(C)** UMI counts and genes detected per nucleus by adipocyte subpopulations in this study and Emont et al. **(D)** Bar graphs displaying the relative proportions of adipocyte subpopulations identified in this study and Emont et al.

bioRxiv preprint doi: https://doi.org/10.1101/2024.04.08.588525; this version posted August 12, 2024. The copyright holder for this preprint (which was not certified by peer review) is the author/funder, who has granted bioRxiv a license to display the preprint in perpetuity. It is made available under aCC-BY 4.0 International license.

**Table S2. Sequences of primers used for quantitative real time PCR (qRT-PCR).**

| Gene | Sequences (Forward) | Sequences (Reverse) |
|---|---|---|
| *Adipoq* | TGTTCCTCTTAATCCTGCCCA | CCAACCTGCACAAGTTCCCTT |
| *Cebpa* | CTGAGAGCTCCTTGGTCAAG | CGAAACCATCCTCTGGGTC |
| *Dlk1* | CTTTCGGCCACAGCACCTAT | CATGGCACCTGCAGACATTG |
| *Emr1* | CTTTGGCTATGGGCTTCCAGTC | GCAAGGAGGACAGAGTTTATCGTG |
| *Fabp4* | AAGGTGAAGAGCATCATAACCCT | TCACGCCTTTCATAACACATTCC |
| *Fam214a* | AGGCTTCCAGTATGGTTTTCAG | AGGCCAAAGAGACCAAGAAC |
| *Pecam1* | CTGCCAGTCCGAAAATGGAAC | CTTCATCCACTGGGGCTATC |
| *Plin1* | AATGAGTTGGCCTGCAGAG | AGGCGGGTAGAGATGGTG |
| *Pparg* | GTGCCAGTTTCGATCCGTAGA | GGCCAGCATCGTGTAGATGA |
| *Vcam1* | CTGGGAAGCTGGAACGAAGT | GCCAAACACTTGACCGTGAC |
| *36B4* | GAGGAATCAGATGAGGATATGGGA | AAGCAGGCTGACTTGGTTGC |
