# Supplementary material for: Robust single nucleus RNA sequencing reveals depot-specific cell population dynamics in adipose tissue remodeling during obesity
Source: bioRxiv. 2024 Apr 8:2024.04.08.588525. Preprint. [Version 1] doi: 10.1101/2024.04.08.588525 (PMC11030456; doi:10.1101/2024.04.08.588525)
Supplement: Supplementary file 1 [file NIHPP2024.04.08.588525v1-supplement-1.pdf]

bioRxiv preprint doi: https://doi.org/10.1101/2024.04.08.588525; this version posted April 8, 2024. The copyright holder for this preprint (which was not certified by peer review) is the author/funder, who has granted bioRxiv a license to display the preprint in perpetuity. It is made available under aCC-BY 4.0 International license.

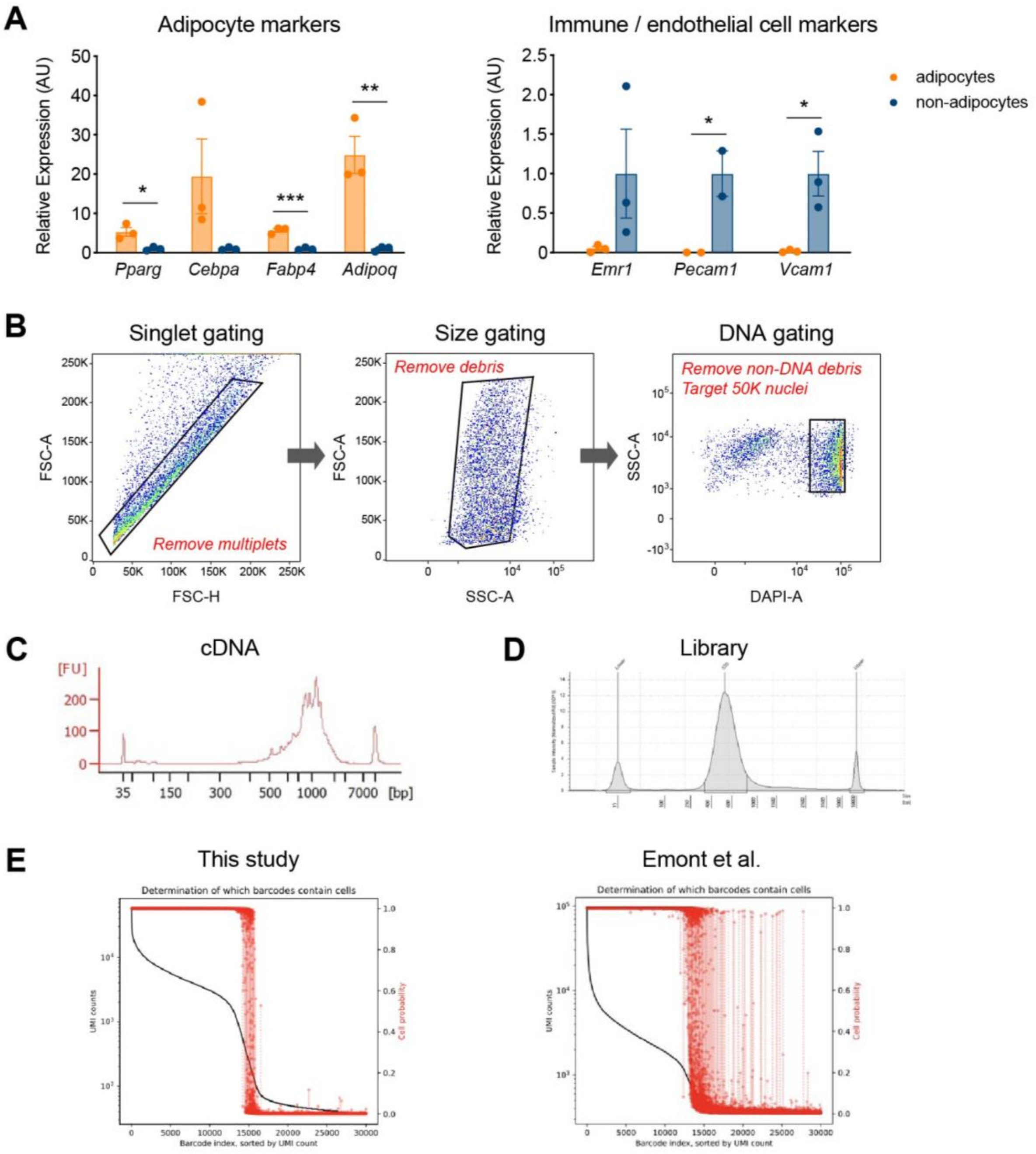

Figure S1. Precise and robust gene expression profiling by our optimized snRNA-seq protocol. (A) Gene expression analysis of adipocyte and immune/endothelial cell markers in adipocyte and non-adipocyte nuclei (*n*=3, each) from eWAT of NuTRAP mice, utilizing the optimized protocol with VRC. Data are presented as mean ± SEM, and statistical analysis was performed using Student's *t* test. * p <0.05, ** p <0.01, *** p <0.001. **(B)** Sequential gating strategies for fluorescence-activated nucleus sorting (FANS) used to sort all Hoechst-positive

bioRxiv preprint doi: https://doi.org/10.1101/2024.04.08.588525; this version posted April 8, 2024. The copyright holder for this preprint (which was not certified by peer review) is the author/funder, who has granted bioRxiv a license to display the preprint in perpetuity. It is made available under aCC-BY 4.0 International license.

nuclei from adipose tissue for snRNA-seq. **(C-D)** Exemplary profiles of **(C)** cDNA and **(D)** sequencing library fragment size distribution, assessed by Agilent Bioanalyzer and TapeStation, respectively. **(E)** Levels of ambient RNA in the samples of this study (left) and Emont et al. (GSE176171, right), examined using CellBender. The plots describe both the rank-ordered UMI counts per droplet (represented by a black line) and the probabilities of each droplet containing a cell (indicated by red dots with dashed lines).

bioRxiv preprint doi: https://doi.org/10.1101/2024.04.08.588525; this version posted April 8, 2024. The copyright holder for this preprint (which was not certified by peer review) is the author/funder, who has granted bioRxiv a license to display the preprint in perpetuity. It is made available under aCC-BY 4.0 International license.

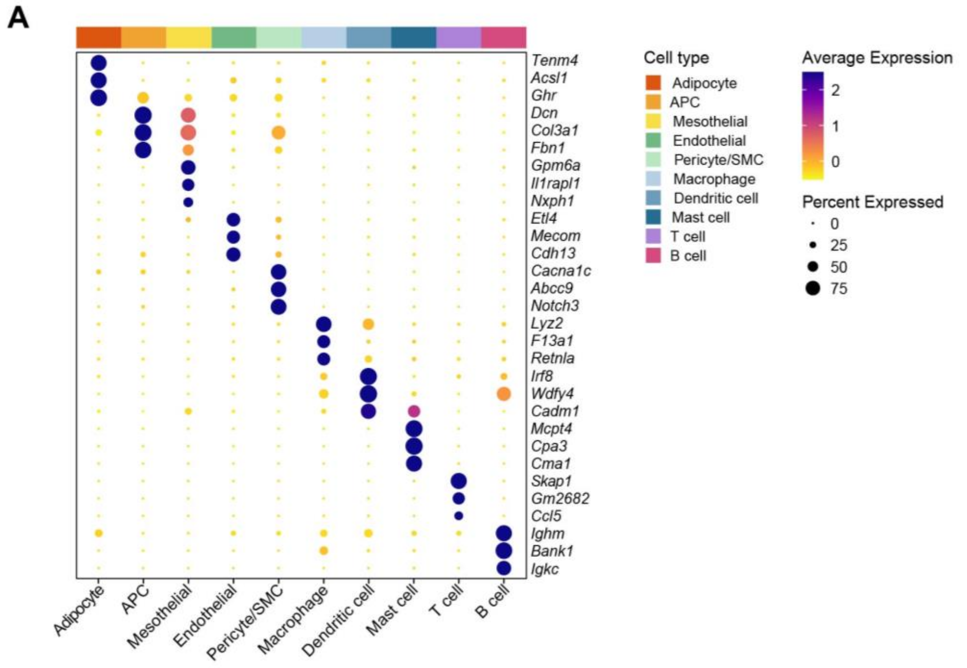

B

	This study	Emont et al.	Sávári et al.
	nuclei no. (%)	nuclei no. (%)	nuclei no. (%)
Adipocyte	10760 (20.2)	39934 (21.7)	4604 (24.3)
APC	14061 (26.3)	51227 (27.8)	5172 (27.3)
Mesothelial	1982 (3.7)	14947 (8.1)	790 (4.2)
Endothelial	6747 (12.6)	6772 (3.7)	NA
Pericyte/SMC	1830 (3.4)	860 (0.5)	NA
Macrophage	15505 (29.0)	65502 (35.5)	7618 (40.3)
Dendritic cell	359 (0.7)	1572 (0.9)	737 (3.9)
Mast cell	181 (0.3)	311 (0.2)	NA
T cell	1361 (2.6)	1960 (1.1)	NA
B cell	609 (1.14)	1202 (0.7)	NA
Total	53395 (100)	184287 (100)	18921 (100)

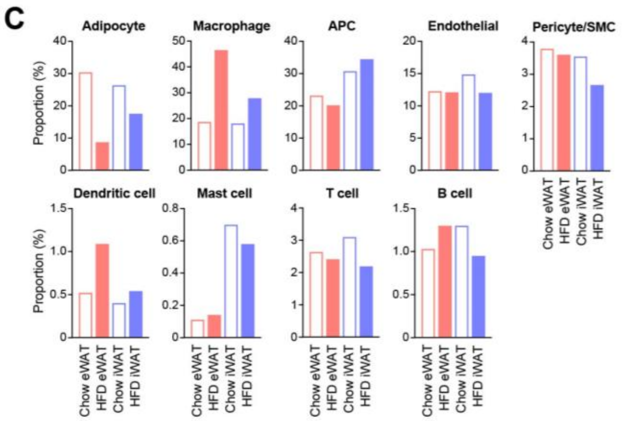

Figure S2. Profile of our single nucleus atlas encompassing diverse cell populations. (A) Dot plots showing average scaled expression of the top three cell type marker genes in each cell population in mouse adipose tissue. **(B)** Comparison of this study with previous snRNA-seq studies regarding the numbers and proportions of recovered nuclei for each cell type. **(C)** Bar graphs showing the relative proportions of individual cell types, split by fat depot and diet.

bioRxiv preprint doi: https://doi.org/10.1101/2024.04.08.588525; this version posted April 8, 2024. The copyright holder for this preprint (which was not certified by peer review) is the author/funder, who has granted bioRxiv a license to display the preprint in perpetuity. It is made available under aCC-BY 4.0 International license.

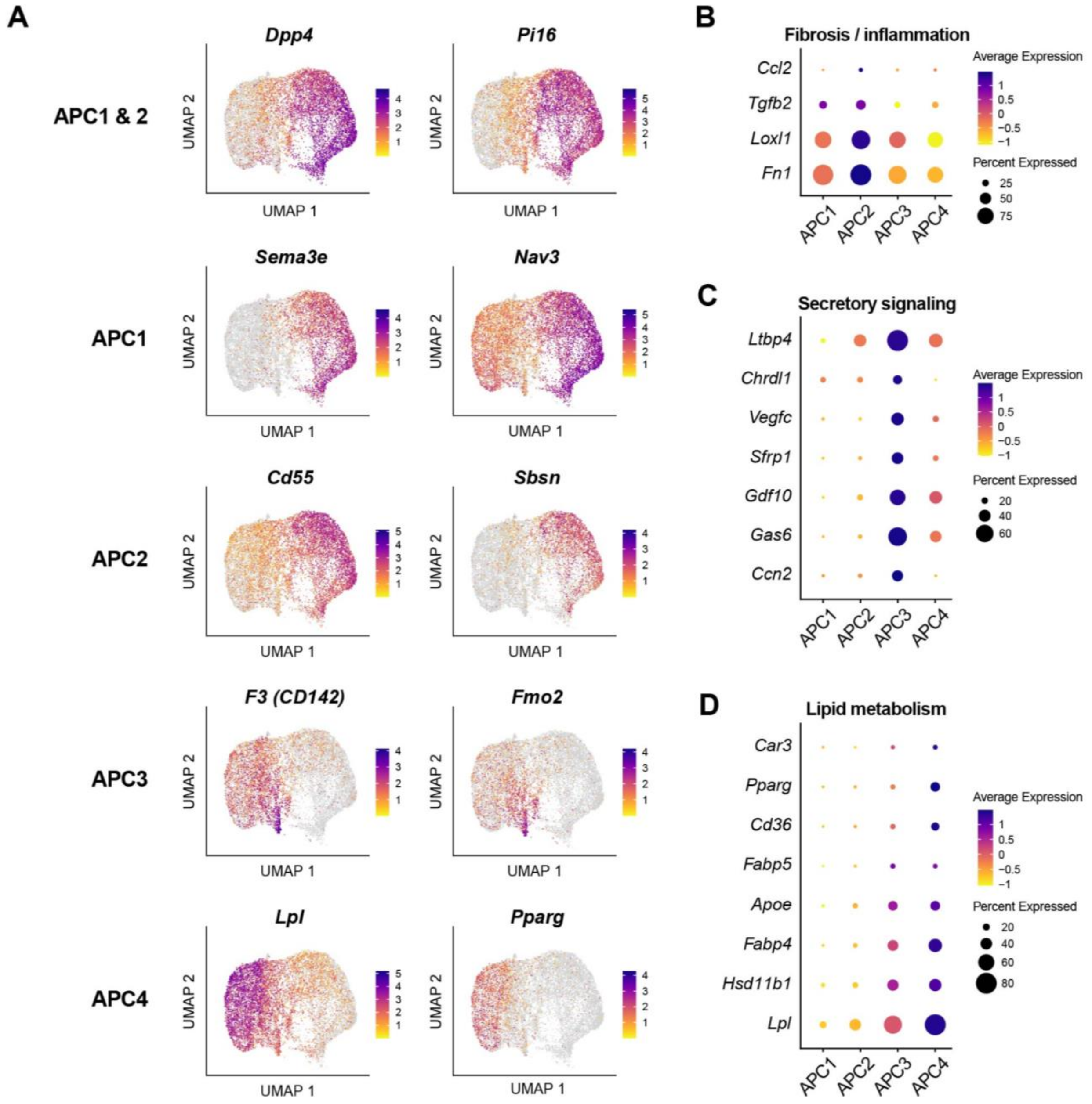

Figure S3. Gene expression profiles of APC subpopulations indicating distinct functional states. (A) UMAP visualization of marker genes for APC subpopulations. **(B-D)** Dot plots showing the expression of genes associated with fibrosis/inflammation **(B)**, secretory signaling **(C)**, and lipid metabolism **(D)** across APC subpopulations.

bioRxiv preprint doi: https://doi.org/10.1101/2024.04.08.588525; this version posted April 8, 2024. The copyright holder for this preprint (which was not certified by peer review) is the author/funder, who has granted bioRxiv a license to display the preprint in perpetuity. It is made available under aCC-BY 4.0 International license.

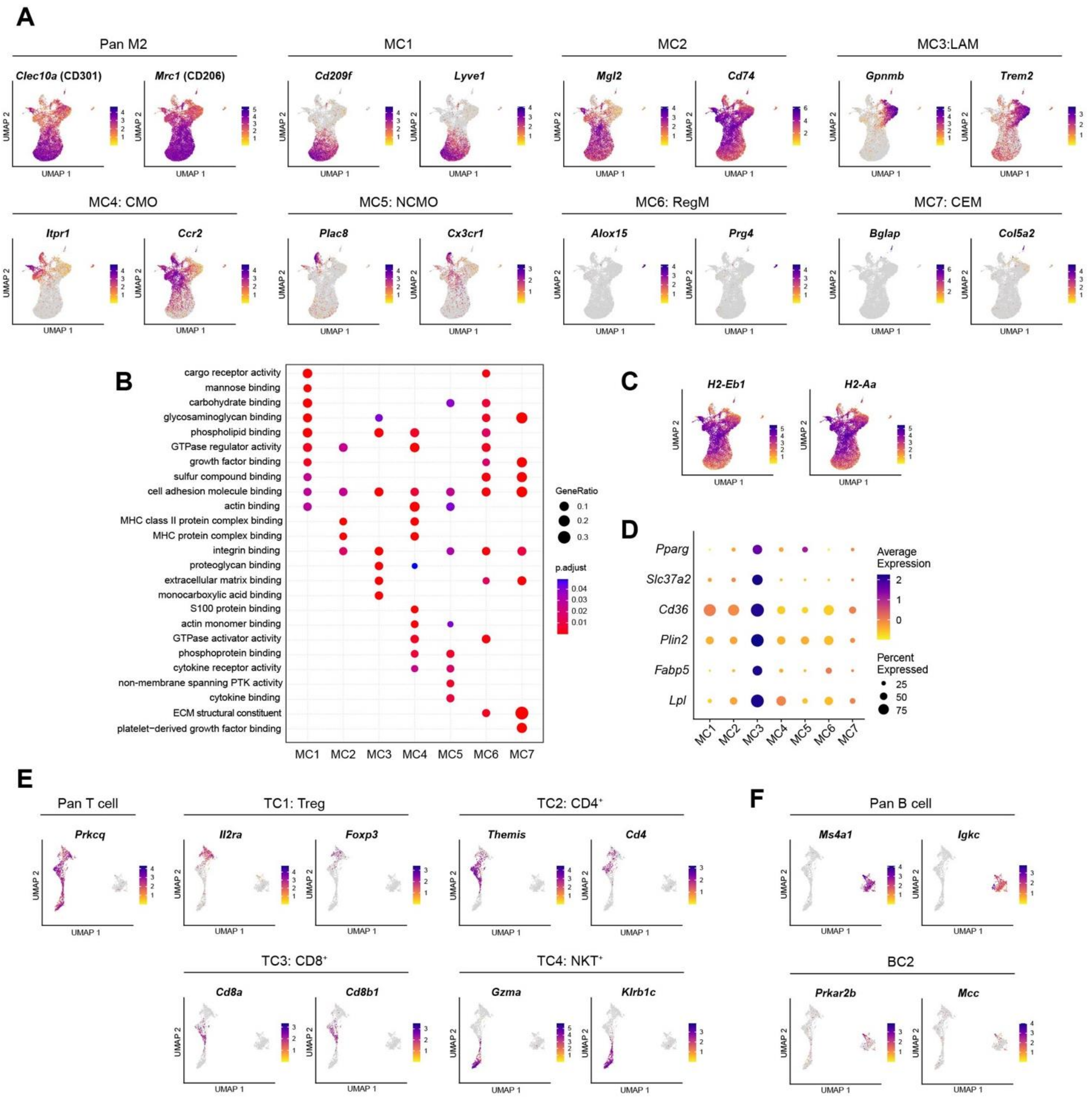

Figure S4. Characterization of diverse immune cell subpopulations. (A) UMAP visualization of marker genes for macrophage/monocyte subpopulations. **(B)** Dot plot illustrating pathways enriched within the top 100 marker genes of macrophage/monocyte subpopulations, as determined by clusterProfiler. **(C)** UMAP visualization of major histocompatibility complex (MHC) genes in macrophage/monocyte subpopulations. **(D)** Dot plot showing the expression of genes associated with lipid metabolism across macrophage/monocyte subpopulations. **(E)** UMAP visualization of marker genes for lymphocyte subpopulations.

bioRxiv preprint doi: https://doi.org/10.1101/2024.04.08.588525; this version posted April 8, 2024. The copyright holder for this preprint (which was not certified by peer review) is the author/funder, who has granted bioRxiv a license to display the preprint in perpetuity. It is made available under aCC-BY 4.0 International license.

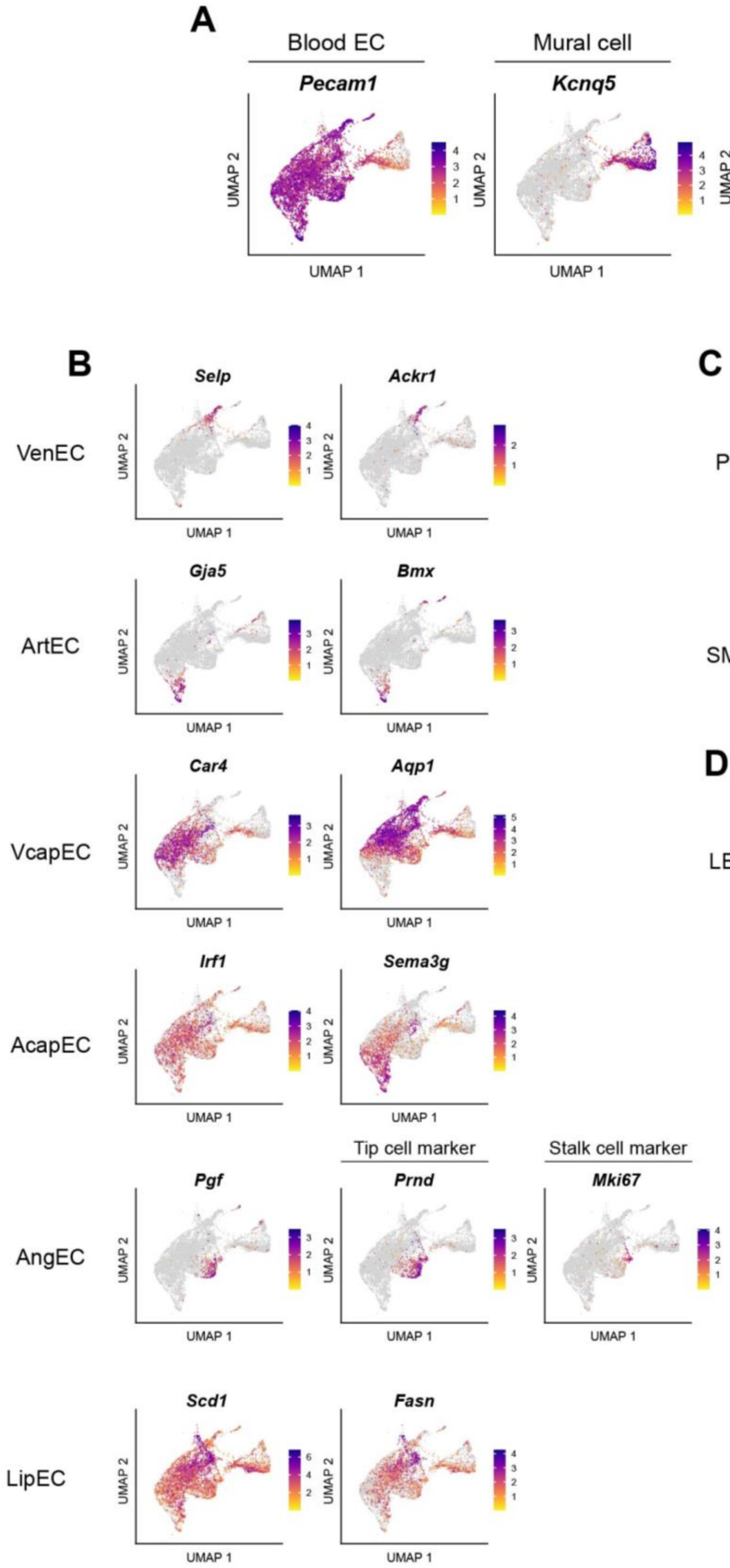

bioRxiv preprint doi: https://doi.org/10.1101/2024.04.08.588525; this version posted April 8, 2024. The copyright holder for this preprint (which was not certified by peer review) is the author/funder, who has granted bioRxiv a license to display the preprint in perpetuity. It is made available under aCC-BY 4.0 International license.

Figure S5. Characterization of diverse vascular cell subpopulations. (A) UMAP visualization using *Pecam1* for blood EC, *Kcnq5* for mural cells, and *Prox1* for LEC within vascular cells. **(B-D)** UMAP visualization of marker genes for **(B)** blood EC subpopulations. **(C)** mural cell subpopulations, and **(D)** LEC subpopulation.

bioRxiv preprint doi: https://doi.org/10.1101/2024.04.08.588525; this version posted April 8, 2024. The copyright holder for this preprint (which was not certified by peer review) is the author/funder, who has granted bioRxiv a license to display the preprint in perpetuity. It is made available under aCC-BY 4.0 International license.

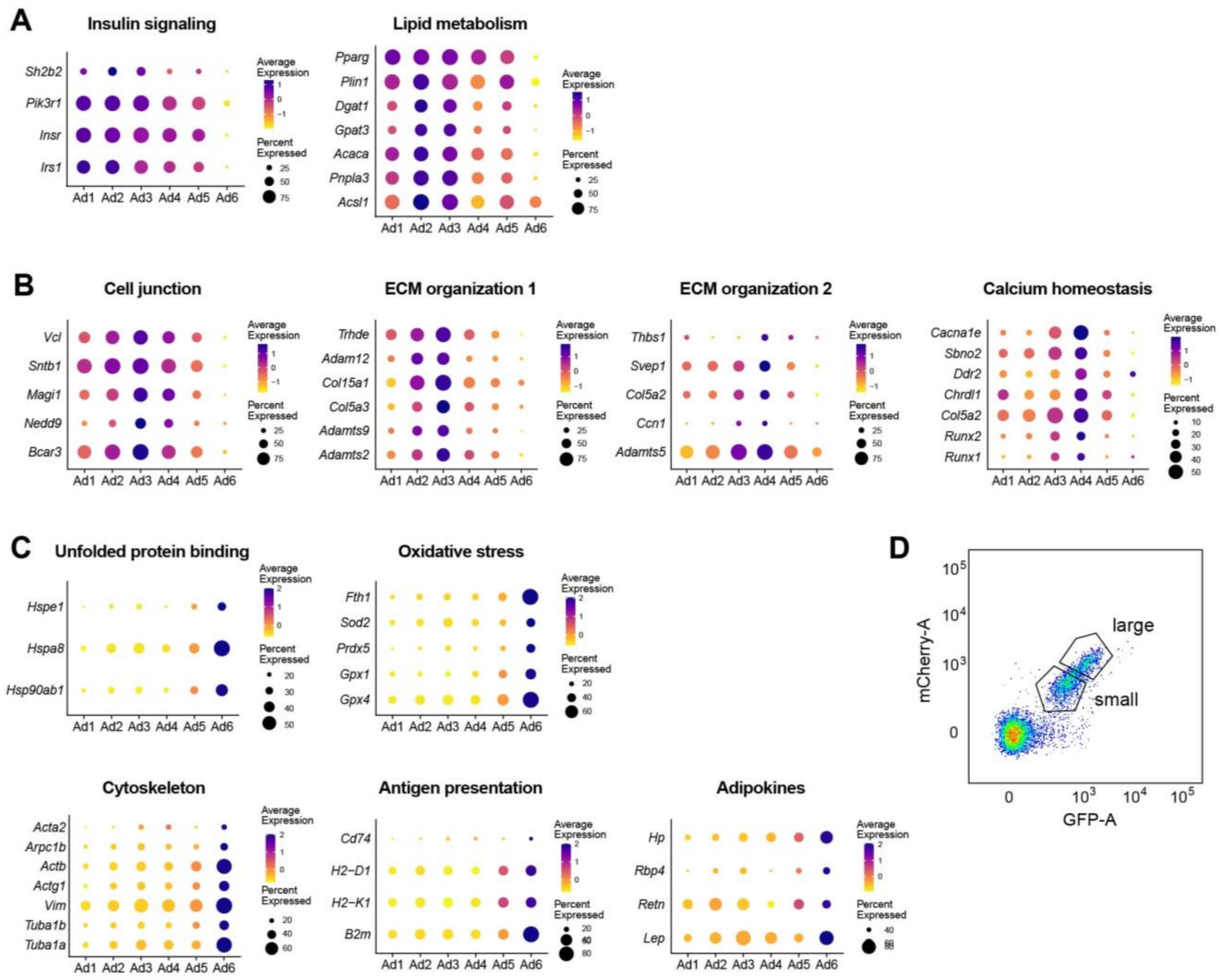

Figure S6. Identification of functional attributes of adipocyte subpopulations. (A-C) Dot plots representing the expression of genes linked to distinct biological processes enriched in different adipocyte subpopulations: **(A)** for Ad1-2, **(B)** for Ad3-4, and **(C)** for Ad6. **(D)** Sorting criteria for gating adipocyte nuclei isolated from iWAT of HFD-fed NuTRAP mice into small and large nuclei.

bioRxiv preprint doi: https://doi.org/10.1101/2024.04.08.588525; this version posted April 8, 2024. The copyright holder for this preprint (which was not certified by peer review) is the author/funder, who has granted bioRxiv a license to display the preprint in perpetuity. It is made available under aCC-BY 4.0 International license.

Table S2. Sequences of primers used for quantitative real time PCR (qRT-PCR).

Gene	Sequences (Forward)	Sequences (Reverse)
Adipoq	TGTTCCTCTTAATCCTGCCCA	CCAACCTGCACAAGTTCCCTT
Cebpa	CTGAGAGCTCCTTGGTCAAG	CGAAACCATCCTCTGGGTC
Dlk1	CTTTCGGCCACAGCACCTAT	CATGGCACCTGCAGACATTG
Emr1	CTTTGGCTATGGGCTTCCAGTC	GCAAGGAGGACAGAGTTTATCGTG
Fabp4	AAGGTGAAGAGCATCATAACCCT	TCACGCCTTTCATAACACATTCC
Fam214a	AGGCTTCCAGTATGGTTTTCAG	AGGCCAAAGAGACCAAGAAC
Pecam1	CTGCCAGTCCGAAAATGGAAC	CTTCATCCACTGGGGCTATC
Plin1	AATGAGTTGGCCTGCAGAG	AGGCGGGTAGAGATGGTG
Pparg	GTGCCAGTTTCGATCCGTAGA	GGCCAGCATCGTGTAGATGA
Vcam1	CTGGGAAGCTGGAACGAAGT	GCCAAACACTTGACCGTGAC
36B4	GAGGAATCAGATGAGGATATGGGA	AAGCAGGCTGACTTGGTTGC
